# Classroom interaction and second language grit as predictors of college English achievement: the mediating role of learners’ foreign language emotions

**DOI:** 10.3389/fpsyg.2026.1797140

**Published:** 2026-03-27

**Authors:** Qiangwei Lu, Yujia Du, Chenglong Zhai, Wenjie Liu, Juan Xu

**Affiliations:** School of Education, Huaibei Institute of Technology, Huaibei, China

**Keywords:** classroom interaction, college English achievement, foreign language boredom, foreign language enjoyment, foreign language anxiety, second language grit

## Abstract

**Introduction:**

Grounded in the Three Pillars framework of positive psychology, this study investigates how classroom interaction (CI) and second language (L2) grit relate to college English achievement (CEA), with a particular focus on the mediating roles of foreign language (FL) emotions. Although prior research has examined these variables separately, the mechanisms linking contextual, dispositional, and emotional factors to achievement remain underexplored.

**Methods:**

A total of 649 second-year non-English-major undergraduates from three universities in eastern China participated in this study. Data were collected using validated scale instruments measuring CI, L2 grit, and FL emotions (enjoyment, anxiety, and boredom), along with students’ comprehensive English course scores. Path analysis in Structural equation modeling and mediation analysis were employed to examine the hypothesized relationships.

**Results:**

The results indicate that neither CI nor L2 grit exerts a significant direct effect on CEA. However, both variables demonstrate significant indirect effects through FL emotions. Specifically, FL enjoyment positively predicts CEA and fully mediates the relationships between CI and CEA and between L2 grit and CEA. In contrast, FL boredom negatively predicts CEA and also serves as a significant mediator. FL anxiety shows no significant predictive or mediating effect.

**Discussion:**

These findings highlight the pivotal role of emotional experiences in linking classroom context and learner traits to academic achievement. They suggest that enhancing positive emotions and reducing boredom may be more effective than focusing solely on interaction or perseverance. The study extends the explanatory power of the Three Pillars framework and offers pedagogical implications for emotion-oriented English teaching in tertiary education.

## 1 Introduction

Recent research and practice in foreign language (FL) education indicate a shift in English teaching objectives from an exclusive emphasis on linguistic knowledge transmission to a broader focus on learners’ holistic development and learning experiences. An expanding body of literature demonstrates that language learning constitutes a highly contextualized, affective, and socialized complex system, rather than solely a cognitive process (Li et al., 2024b). Learners’ emotional experiences, motivation, and level of engagement in the classroom are closely associated with the effectiveness of language input, the quality of output, and the adoption and sustainability of learning strategies (Dewaele and Li, 2020).

Despite these developments, undergraduate English teaching in many universities remains strongly test-oriented due to the persistent dominance of evaluation systems focused on examination scores, standardized test pass rates (e.g., CET-4 and CET-6), and course grade point averages. Within such contexts, instructional practices and learner strategies often prioritize test preparation and discrete knowledge explanation (Wang and Marecki, 2021; Zhang, 2025). Consequently, the depth and quality of teacher-student and student–student interactions are often limited, and learners’ agency, participation, and emotional experiences are insufficiently addressed. This outcome-oriented instructional model, which downplays process-oriented learning experiences, may undermine learners’ intrinsic interest in English, suppress their willingness to participate in classroom activities, and, over time, induce negative emotions (Pekrun, 2006; Li et al., 2024a), thereby constraining sustained engagement and overall learning effectiveness.

From a theoretical perspective, positive psychology (PP) offers an important lens through which to reconsider the role of affective factors in FL classrooms. In particular, drawing on the Three Pillars framework in PP, MacIntyre (2016) argues that FL learning outcomes are not determined solely by cognitive ability or linguistic input, but rather emerge from the joint influence of positive individual traits, positive emotional experiences, and supportive learning environments. Among these components, the classroom environment, particularly the quality of interaction, has been identified as critical for fostering positive emotions and sustaining long-term learning engagement (Zhang et al., 2025). Nevertheless, traditional second language acquisition (SLA) research has often examined individual differences such as motivation, anxiety, or learning styles as relatively stable learner traits within de-contextualized frameworks (Teimouri et al., 2022). Consequently, limited attention has been paid to learners’ dynamic interactions with teachers, peers, and learning tasks in specific classroom contexts, and the mechanisms through which individual and environmental factors interact remain insufficiently explored (Li et al., 2024b). In this sense, classroom interaction (CI) should not be viewed merely as an instructional technique or classroom management strategy, but rather as a core psychological and contextual variable warranting systematic investigation in relation to FL learning outcomes.

In addition, second language (L2) grit is an important extension of PP in the field of SLA. It has attracted increasing scholarly attention in recent years. L2 grit is closely associated with learners’ long-term engagement, persistence behaviors, and academic performance. It may also influence learning processes and outcomes by shaping how learners perceive classroom challenges and emotional experiences (e.g., Lee, 2020; Elahi Shirvan et al., 2021; Derakhshan and Fathi, 2024; Wu et al., 2024). However, existing research has mainly investigated the direct effects of grit on achievement. There is limited empirical evidence on how grit operates in relation to CI and FL emotions, especially in Chinese university English classrooms.

In light of these considerations, this study adopts a PP framework. It takes CI as a key contextual variable to examine the possible relation between CI and L2 grit on college English achievement (CEA) among university students. FL emotions are incorporated to explore the mediating roles of FL enjoyment, FL anxiety, and FL boredom in this relationship. This study aims to clarify how cognitive, affective, and contextual factors work together in university English learning. It seeks to provide theoretical support and empirical evidence for optimizing CI design, fostering positive emotional climates, and sustaining learner engagement in Chinese tertiary English education. The study also extends the explanatory range of PP in SLA research.

## 2 Literature review

### 2.1 Three pillars in PP

PP centers on promoting holistic individual development and enhancing personal well-being (Seligman, 2001). Within this framework, Seligman and Csikszentmihalyi (2000) propose the Three Pillars theory, which posits that the development and well-being of an individual rely on the synergistic interaction of three key elements: positive individual traits (e.g., personality characteristics), positive subjective experiences (e.g., positive emotions), and positive institutions (e.g., supportive groups and environments). Specifically, learners with positive individual traits are more likely to hold constructive perceptions and evaluations of their surroundings, while those situated in supportive and positive environments are more likely to experience positive emotions. These positive emotions, in turn, reinforce adaptive interactions between individuals and their environment, thereby continuously facilitating personal growth and skill development.

Building on this framework, MacIntyre (2016) applies the Three Pillars theory to the field of SLA. He emphasizes that positive individual traits, emotional experiences, and language learning environments interact and mutually reinforce each other. Together, they jointly shape FL learning outcomes. However, existing empirical research has primarily focused on individual differences among learners. There is relatively limited attention to the environmental dimension. In particular, there remains a lack of systematic examination of how the dynamic interplay among the three pillars in FL learning contexts predicts academic achievement (Budzińska and Majchrzak, 2021; Derakhshan and Fathi, 2024). Academic achievement is a central concern for students, teachers, and parents. Thus, investigating this relationship from a PP perspective holds both theoretical and practical significance.

Based on the above theoretical rationale and research gaps, this study employs the Three Pillars framework. It examines the predictive roles of CI, individual traits (grit), and FL emotions on CEA. Notably, CI is conceptualized as a concrete representation of the “positive institutions” pillar. This reflects the specific realities of the Chinese FL learning context. For undergraduate FL learners, classroom activities are the main arena for language learning. The quality of CI plays a direct and crucial role in shaping both learning experiences and academic outcomes (Wang and Castro, 2010). From a process-oriented perspective, CI and L2 grit are relatively stable environmental and trait-based predictors (Wu et al., 2024). However, academic achievement depends on learners’ moment-to-moment allocation of cognitive resources, task persistence, and engagement behaviors (Zhang and Zhang, 2023). The broaden-and-build theory (Fredrickson, 2003) proposes that positive emotions broaden attentional scope and enhance cognitive flexibility. These emotions also help build psychological resources, which facilitate sustained engagement and deeper processing. By contrast, negative emotions like boredom narrow attention and reduce motivation. Therefore, CI and L2 grit do not directly translate into academic achievement. They first shape learners’ emotional experiences, which then regulate cognitive engagement and behavioral persistence (Li et al., 2021). In this sense, FL emotions are a proximal pathway linking environmental and dispositional factors to achievement outcomes.

### 2.2 Classroom interaction (CI)

CI refers to the reciprocal actions and influences that occur between teachers and students, as well as among students, within specific educational contexts during the teaching and learning process. It goes beyond knowledge transmission by involving behavioral feedback, emotional exchange, and psychological changes. Educational research has highlighted the multi-perspective nature of CI (e.g., Kim and Cappella, 2016). Previous studies have categorized interaction based on participants and behavioral roles; for example, it has been broadly classified into teacher-student interaction and student–student interaction (Luckner and Pianta, 2011; Moskowitz et al., 2022). CI is a dynamic, multidimensional process, and it includes cognitive dimensions such as shared thinking, affective dimensions such as psychological empathy, behavioral dimensions such as co-constructed attitudes, and value-oriented dimensions like shared meaning (Wang and Castro, 2010; Wang and Marecki, 2021). The primary purpose of CI is to evoke students’ emotional resonance, influence their behaviors, and contribute to the formation of their social identity (Dan and Bai, 2025). Through collaborative participation in learning activities, teachers and students create a shared learning environment (Han and Ellis, 2021; Ma and Wei, 2022). They achieve learning goals through knowledge exchange and support holistic student development. CI enhances students’ understanding, stimulates emotional engagement, and fosters critical thinking and social skills (Ma and Wei, 2022; Dan and Bai, 2025).

In this study, CI refers to the reciprocal communication and engagement between teachers and students, as well as among students within a specific instructional context. It involves both verbal and non-verbal behaviors, along with emotional changes. These interactions aim to achieve specific learning objectives. CI includes activities such as answering questions, classroom discussions, group work, and role-playing, as well as knowledge transmission, emotional resonance, behavioral adjustment, and opinion sharing. To further investigate the relationship between CI and CEA in the college context, this study uses the Three Pillars theory to examine the predictive role of CI on CEA and the mediating roles of FL emotions.

### 2.3 L2 grit

Grit refers to an individual’s perseverance and passion in pursuing long-term goals (Duckworth et al., 2007), and it is generally conceptualized as comprising two components: perseverance of effort and consistency of interests. Duckworth and Quinn (2009) argue that grit is as important as innate ability in influencing academic achievement. Even after controlling for ability-related factors, grit still demonstrates incremental predictive power for academic outcomes (Tang X. et al., 2021). Because of its significant impact on academic achievement, grit has increasingly been applied to the SLA domain (Teimouri et al., 2022). Empirical findings suggest that learners with higher levels of L2 grit tend to possess clearer learning goals. They also invest more time and effort in language study, demonstrate greater self-efficacy regarding their FL abilities, and exhibit higher academic achievement and well-being (e.g., Teimouri et al., 2022; Zhang, 2023).

However, despite the recognized value of grit in FL learning, its empirical influence on language achievement remains inconsistent. Several studies have reported significant positive correlations between L2 grit and English achievement (e.g., Lee, 2020; Alamer, 2021; Wang, 2023; Calafato, 2025), whereas Khajavy et al. (2021) find no significant association between L2 grit, its two components, and academic achievement. Consequently, the relationship between L2 grit and language achievement remains unclear, and whether grit’s predictive effect on FL academic outcomes is moderated by other factors requires further investigation. This inconsistency suggests that the influence of L2 grit on academic achievement may not always operate through a direct pathway (Wu et al., 2024). While grit reflects learners’ long-term persistence and commitment to language learning, its effectiveness in promoting achievement may depend on how such perseverance translates into actual engagement during learning activities. In particular, learners’ emotional experiences in language classrooms may play an important role in shaping whether sustained effort results in effective learning behaviors and performance (Liu et al., 2024). Therefore, examining emotional factors may help explain why the relationship between L2 grit and FL achievement varies across empirical studies.

### 2.4 FL emotions

Traditional research in SLA has primarily followed a purely cognitive approach, and it often neglects the important role of non-cognitive factors like emotions (Li et al., 2024b). Emotions appear to play a moderating role in language learning. Increasingly, scholars recognize the diversity and functions of emotions in this process (Dewaele and Li, 2020; Lu, 2025). With the rise of PP, attention has shifted toward positive emotions, especially enjoyment (Dewaele and MacIntyre, 2014). FL enjoyment in SLA, as a positive, high-arousal, and process-oriented emotion, can broaden learners’ cognitive and behavioral resources, thereby exerting a beneficial effect on academic achievement (Li et al., 2018; Li C. C., 2025). In contrast, FL anxiety is typically considered a process-oriented emotion with a detrimental effect on FL learning (Teimouri et al., 2019; Shen et al., 2026).

In recent years, FL boredom has emerged as a new focal point in emotion research. FL boredom generally arises when learners perceive low value in academic tasks or lack a sense of control, leading to attentional disengagement and emotional fluctuation. Consequently, FL boredom has been found to be negatively associated with learning motivation, cognitive resource allocation, and learning outcomes (e.g., Macklem, 2015; Li et al., 2022; Li and Li, 2024). Empirical studies have reported that FL enjoyment is positively associated with FL learning behaviors and academic achievement (Teimouri et al., 2019; Shen et al., 2026), whereas FL anxiety and FL boredom tend to be negatively associated with academic achievement (Teimouri et al., 2019).

Although numerous studies have established the direct role of FL emotions as learner-internal factors in influencing FL achievement, their mediating or moderating functions in the relationships between external factors (e.g., CI), individual traits (e.g., L2 grit), and academic outcomes remain underexplored and warrant systematic investigation.

### 2.5 CI, L2 grit, FL emotions, and SLA

Based on the Three Pillars in PP, the mechanisms that facilitate FL learning can be seen as the result of interactions among the external environment, individual traits, and FL emotions. Learners with higher levels of L2 grit and resilience tend to perceive CI more positively (Li G. C., 2025), and situated in high-quality interactive classroom environments are more likely to experience positive emotions. Recent studies further indicate that FL enjoyment may arise from multiple classroom-related sources, including teacher support, peer interaction, and engaging learning activities (e.g., Wang and Marecki, 2021; Wang et al., 2024). These positive emotions may further strengthen learners’ engagement with the learning environment and are associated with FL academic development (Zhang et al., 2025). Nevertheless, the direct relationship between classroom interaction and academic achievement has not always been consistently supported in previous research. While some studies report that interactive classroom environments facilitate deeper learning and improved academic outcomes (e.g., Lesiana et al., 2024; Dan and Bai, 2025), others suggest that the effectiveness of interaction may depend on learners’ psychological experiences (e.g., motivation, emotions) during classroom participation (e.g., Joe et al., 2017; Wei et al., 2024). In other words, interaction itself may not automatically translate into improved achievement unless it generates meaningful cognitive and emotional engagement among learners.

L2 Grit is a key individual trait, closely tied to learners’ emotional experiences and external factors such as CI and peer relationships. Grit enables learners to respond more proactively to environmental influences within CI, as a positive interactional climate can activate learners’ positive traits and enhance their cognitive engagement during lessons (Seligman and Csikszentmihalyi, 2000; Teimouri et al., 2021). In turn, the positive or negative characteristics of the CI climate may moderate the relationship between grit, FL achievement, and the experience of enjoyment (Wei et al., 2019).

These considerations suggest a close interrelationship among CI, L2 grit, and FL emotions. However, whether CI and L2 grit can elicit positive emotions, suppress negative ones, and contribute to better FL academic achievement remains an open question. This uncertainty further suggests that FL emotions may function as an important psychological mechanism linking environmental and individual factors with learning outcomes. Therefore, this highlights the need for further empirical investigation. FL emotions, as learner-internal psychological experiences, likely drive motivation, engagement, and learning-well being directly (Dewaele, 2022; Wang et al., 2024; Lu, 2025). Treating FL emotions separately from CI and L2 grit may hinder a full understanding of the mechanisms behind differences in FL learning outcomes.

In short, positive individual traits such as L2 grit can help learners maintain sustained interest and persistent effort in FL learning, while positive emotions, such as enjoyment, can broaden cognitive and behavioral resources, regulate negative emotions (e.g., anxiety and boredom), and enhance learning engagement (Li, 2024). A supportive, interactive classroom environment further fosters the development of positive traits and emotions (Wang et al., 2024).

### 2.6 Summary

Although prior studies have examined relationships among classroom climate, grit, emotions, and academic achievement, mainstream studies have focused on isolated links. For example: grit leading to achievement, classroom climate influencing emotions, or emotions affecting achievement (e.g., Tang D. J. Z. et al., 2021; Wei et al., 2024; Li G. C., 2025; Zhang et al., 2025). Previous research often treated emotions as parallel predictors. They were not considered as proximal psychological mechanisms that transmit the influence of contextual and trait-based factors onto achievement outcomes (Camacho-Morles et al., 2021). In addition, empirical attention has centered mostly on enjoyment and anxiety. Boredom, despite its increasing theoretical relevance, has been less systematically examined.

This study advances existing research in three ways. First, it proposes and tests a hierarchical process model grounded in the Three Pillars framework, explicitly positioning FL emotions as mediating mechanisms linking CI and L2 grit to achievement. Second, it differentiates among enjoyment, anxiety, and boredom within the same analytical framework, allowing for comparative evaluation of their mediating roles. Third, it situates the model within the context of Chinese tertiary English education, characterized by standardized curricula and examination-oriented structures, thereby offering specific evidence for the interaction of environmental and emotional predictors.

## 3 Methodology

### 3.1 Research hypothesis

**Figure 1:**
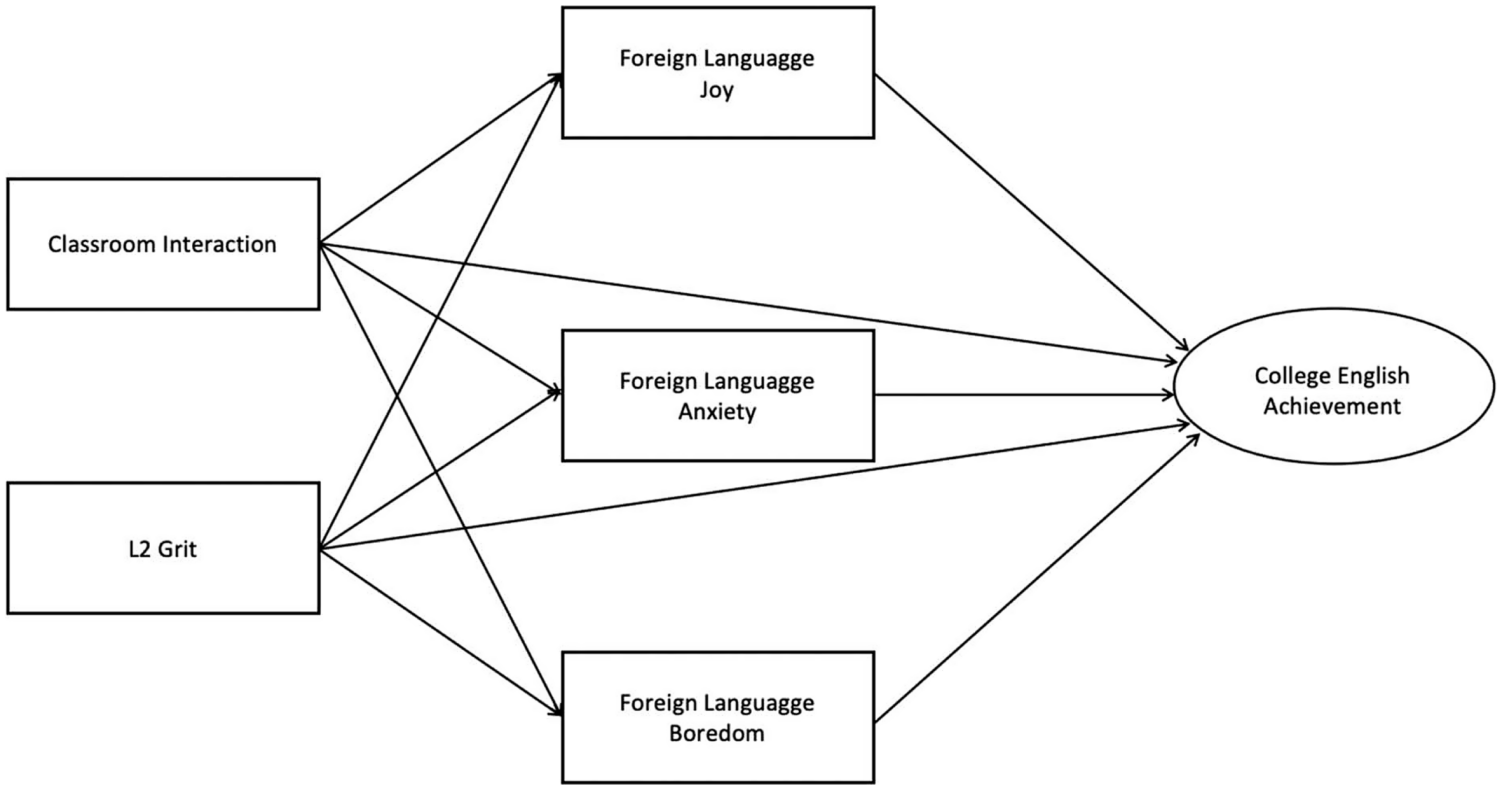
Research hypothesis model.

Based on these considerations, the present study hypothesizes that CI and L2 grit are likely to predict CEA indirectly through FL emotions, including FL enjoyment, FL anxiety, and FL boredom (see Figure 1). The specific hypotheses are as follows:

*Hypothesis 1*: CI and L2 grit play a positive predictive role on CEA.

*Hypothesis 2*: FL emotions (FL enjoyment, FL anxiety, and FL boredom) play a mediating role between CI, L2 grit and CEA.

### 3.2 Participants

After obtaining consent from the universities, teachers, and students, participants were recruited through convenience sampling from three universities (including one normal university, one engineering college and one institution of technology) in two cities of eastern China. A total of 661 questionnaires were returned, and 12 invalid responses were excluded based on repeated items, resulting in an effective response rate of 98.18%. Of the valid sample, 47.25% were male, and 52.75% were female, with ages ranging from 20 to 22 years. Participants had studied English for approximately 15 years, and were engaged in more than 8 h of FL learning per week. They possessed a vocabulary of approximately 3,200 words and could read English novels of moderate difficulty and have simple daily conversations. All participants used the same nationally standardized college English textbook, *New Horizon College English 3*. The curriculum standards and teaching syllabus were uniformly established and administered at the national level. At the time of data collection, all participants received a total of four English lessons per week, with each lesson lasting 45 min.

Participants were recruited from three universities across two cities. Although demographic and institutional differences may act as potential confounders, key background variables (e.g., prior English proficiency, socio-economic status) were not obtained at the time of data collection and thus were not included as covariates. In addition, because the number of universities was small, formal multilevel modeling or cluster-robust estimation was not conducted. To partially mitigate contextual heterogeneity, the participating institutions followed the same nationally standardized curriculum, used the same textbook (*New Horizon College English 3*), and implemented a uniform teaching syllabus. Nevertheless, residual confounding and cluster dependence cannot be fully ruled out.

### 3.3 Research instruments

The questionnaires were distributed and collected using the online questionnaire platform. The survey consisted of two parts. The first part gathered participants’ individual information and their CEA. CEA in this study was operationalized using the comprehensive course score, which incorporated both process-based (40%) and summative (60%) assessments. The process-based component included classroom performance, task completion, and attendance, allowing for the evaluation of students’ English learning over time. The summative examination component was centrally designed within each institution based on the unified syllabus and covered standardized language competencies. Although grading was conducted locally, scoring criteria for written examinations were specified in advance and aligned with institutional marking rubrics. These procedures were intended to enhance comparability across institutions, although minor variations in implementation cannot be entirely excluded. By integrating multiple sources of assessment rather than relying solely on a single final examination, the measure aimed to capture students’ sustained engagement and performance in English learning.

The second part assesses CI, L2 grit, and FL emotions, including FL enjoyment, FL anxiety, and FL boredom. All items were presented in Chinese using a five-point Likert scale.

CI was measured using the Classroom Interaction Scale developed by Dancer and Kamvounias (2005), which consisted of 15 items (Items 3–17) across three dimensions: Instructor Facilitation of Interaction, Student Participation, and CI Climate. The measurement model demonstrated good fit, with χ^2^/df = 2.56, CFI = 0.93, TLI = 0.95, RMSEA = 0.068, and SRMR = 0.037, indicating satisfactory structural validity of the scale. The Cronbach’s alpha and KMO were 0.967 and 0.923, respectively, suggesting high internal consistency.

L2 grit was measured by the L2 Grit Scale developed by Teimouri et al. (2022), which consisted of 9 items (Item 18-Item 26). The measurement model demonstrated good fit, with χ^2^/df = 1.93, CFI = 0.97, TLI = 0.98, RMSEA = 0.070, and SRMR = 0.032, indicating satisfactory structural validity of the scale. The Cronbach’s alpha and KMO were 0.870 and 0.818, respectively, suggesting high internal consistency.

FL enjoyment was chosen to be measured by the Chinese version of the FL Enjoyment Scale translated and validated by Li et al. (2018), which contained 11 items to measure the three sub-dimensions of FL environment enjoyment, FL teacher enjoyment, and FL personal enjoyment (Item 27-Item 37). The measurement model demonstrated good fit, with χ^2^/df = 2.39, CFI = 0.95, TLI = 0.92, RMSEA = 0.063, and SRMR = 0.039, indicating satisfactory structural validity of the scale. The Cronbach’s alpha and KMO were 0.924 and 0.897, respectively, suggesting high internal consistency.

FL anxiety was measured by the FL Anxiety Scale developed by Dewaele and MacIntyre (2014), which contained 8 items (Item 38-Item 45). The measurement model demonstrated good fit, with χ^2^/df = 2.42, CFI = 0.93, TLI = 0.91, RMSEA = 0.076, and SRMR = 0.041, indicating satisfactory structural validity of the scale. The Cronbach’s alpha and KMO were 0.928 and 0.922, respectively, suggesting high internal consistency.

FL boredom was measured by the FL Classroom Boredom Scale developed by Li et al. (2023) and contained 7 items (Item 46-Item 52). The measurement model demonstrated good fit, with χ^2^/df = 2.17, CFI = 0.98, TLI = 0.98, RMSEA = 0.048, and SRMR = 0.029, indicating satisfactory structural validity of the scale. The Cronbach’s alpha and KMO were 0.951 and 0.918, respectively, suggesting high internal consistency.

The results of the construct validity of each scale are shown in Table 1. To establish the convergent validity, AVE and CR were computed. Table 2 presents all AVE values exceeded the recommended cutoff of 0.50, and all CR values were above 0.70, indicating satisfactory convergent validity and internal consistency. These findings suggest that the observed indicators adequately represent their respective latent constructs.

**Table 1:** Construct validity of each scale.

Scales	CFA					*Cronbach α* (value)	Kaiser-Meyer-Olkin measure of sampling adequacy (KMO)
	χ^2^/df	CFI	TLI	RMSEA	SRMR		
CI Scale	2.56	0.93	0.95	0.068	0.037	0.967	0.923
L2 Grit Scale	1.93	0.97	0.98	0.070	0.032	0.870	0.818
FL Enjoyment Scale	2.39	0.95	0.92	0.063	0.039	0.924	0.897
FL Anxiety Scale	2.42	0.93	0.91	0.076	0.041	0.928	0.922
FL Boredom Scale	2.17	0.98	0.98	0.048	0.029	0.951	0.918

**Table 2:** Convergent validity of each scale.

Scales	AVE	CR
CI Scale	0.642	0.922
L2 Grit Scale	0.765	0.865
FL Enjoyment Scale	0.826	0.948
FL Anxiety Scale	0.661	0.877
FL Boredom Scale	0.653	0.921

### 3.4 Data analysis

Firstly, the data were descriptively analyzed using SPSS 26.0 to observe the basic correlation between CI, L2 grit, FL emotions and CEA. Secondly, the hypothesized relationships were examined using path analysis within the structural equation modeling (SEM) framework. Because composite scores were computed for each construct based on validated multi-item scales, all variables in the structural model were treated as observed variables. The structural paths among CI, L2 grit, FL emotions, and CEA were estimated simultaneously using AMOS (21.0). This approach allows for the simultaneous estimation of direct and indirect effects within a single covariance-based model. Besides, the path cases were tested with correlation fit indexes with the following criteria: χ^2^/df less than 3, CFI and TLI greater than 0.9, RMSEA and RSMR less than 0.08 (Wen et al., 2004). Finally, the mediating role of FL emotions was analyzed using mediation analysis by the bootstrapping method, setting 5,000 iterations for repeated sampling test.

## 4 Findings

### 4.1 Overall description of CI, L2 grit, and FL emotions

From the descriptive analysis in Table 2, it can be seen that the average levels of CI, L2 grit, FL enjoyment and anxiety are basically located in the mid-level region, with CI having the highest level. However, the average level of CI boredom is relatively lower than the mid-level region. The results indicated that all variables were significantly associated with CEA, excluding FL anxiety (Table 3).

**Table 3:** Descriptive analysis and correlation analysis (*N* = 649).

Variables	1	2	3	4	5	6
1. CI	—					
2. L2 grit	0.451**	—				
3. FL enjoyment	0.704**	0.574**	—			
4. FL anxiety	−0.027**	0.174**	0.010**	—		
5. FL boredom	−0.239**	0.113**	−0.318**	0.491**	—	
6. CEA	0.100**	0.103**	0.206**	−0.073**	−0.183**	—
Scale	1–5	1–5	1–5	1–5	1–5	0–100
Mean value	3.685	3.240	3.491	3.372	2.760	75.043
Standard deviation	0.678	0.442	0.604	0.746	0.756	10.074

*p < 0.05, **p < 0.01.

### 4.2 Path analysis of CI, L2 grit, FL emotions, and CEA

Path analysis is conducted using AMOS 21.0 to examine the relationships among CI, L2 grit, FL emotions, and CEA. The results are presented in Table 4.

**Table 4:** Path analysis (*N* = 649).

Independent variable → Dependent variable	Standard error	Z (CR)	*p*	Standardized path coefficient *β*
CI → CEA	0.836	−1.814	0.070	−0.103
L2 Grit → CEA	1.134	1.155	0.248	0.057
FL Enjoyment → CEA	0.974	3.439	0.001**	0.202
FL Anxiety → CEA	0.526	−0.480	0.631	−0.019
FL Boredom → CEA	0.546	−3.503	0**	−0.144
CI → FL Enjoyment	0.026	19.479	0**	0.561
L2 Grit → FL Enjoyment	0.040	10.964	0**	0.316
CI → FL Anxiety	0.047	−3.297	0.001**	−0.142
L2 Grit → FL Anxiety	0.074	−5.803	0**	−0.25
CI → FL Boredom	0.046	−9.279	0**	−0.382
L2 Grit → FL Boredom	0.071	−7.478	0**	−0.308

*p < 0.05, **p < 0.01.

First, neither CI nor L2 grit exerts a significant direct effect on CEA (*β* = −0.103, *p* > 0.05; *β* = 0.057, *p* > 0.05, respectively).

Second, among the three FL emotion variables, FL enjoyment has a significant positive effect on CEA (*β* = 0.202, *p* < 0.01), whereas FL anxiety does not significantly predict CEA (*β* = −0.019, *p* > 0.05). In contrast, FL boredom shows a significant negative effect on CEA (*β* = −0.144, *p* < 0.01).

Third, CI significantly and positively predicts FL enjoyment (*β* = 0.561, *p* < 0.01), while exerting significant negative effects on FL anxiety (*β* = −0.142, *p* < 0.05) and FL boredom (*β* = −0.382, *p* < 0.01).

Fourth, L2 grit significantly and positively predicts FL enjoyment (*β* = 0.316, *p* < 0.01), and significantly negatively predicts FL anxiety (*β* = −0.250, *p* < 0.01) and FL boredom (*β* = −0.308, *p* < 0.01).

Based on these results, non-significant paths are removed, and the model is re-estimated. The fit indicators of the revised model indicate an acceptable to good model fit, with χ^2^/df = 2.75, CFI = 0.92, TLI = 0.90, RMSEA = 0.065, SRMR = 0.047 (see Table 5). These results suggest that the proposed path model demonstrates satisfactory fit and that the relationships among the variables are well represented.

**Table 5:** Model fit indicators of CFA.

Fit index	Recommended cutoff	Model value
χ^2^/df	<3.00	2.75
CFI	≥0.90	0.92
TLI	≥0.90	0.90
RMSEA	≤0.08	0.065
SRMR	≤0.08	0.047

(1) Recommended cutoff criteria based on Wen et al. (2004). (2) Estimation method: maximum likelihood (ML) estimation.

### 4.3 Mediation analysis of FL emotions (enjoyment, anxiety, and boredom) between CI, L2 grit, and CEA

The results of the mediation analysis are presented in Table 6. The 95% confidence interval for the indirect effect through FL enjoyment does not include zero (0.032 to 0.200). Besides, both path *a* (*CI → FL enjoyment*) and path *b* (*FL enjoyment → CEA*) are significant, while the direct effect (*c’*) is non-significant. This pattern indicates a full mediation effect, supporting the indirect pathway “CI → FL enjoyment → CEA”. In addition, the indirect effect of CI on CEA via FL boredom is significant, as the 95% confidence interval does not include zero (0.009 to 0.110). This indicates that FL boredom significantly mediates the relationship between CI and CEA, supporting the indirect pathway “CI → FL boredom → CEA”.

**Table 6:** Mediation analysis (*N* = 649).

Item	Coefficient a	Coefficient b	a*b (95% BootCI)	Direct effect c’	Result
CI → FL Enjoyment → CEA	0.499**	3.366**	0.032 ~ 0.200	−1.523	*Full Mediation*
CI → FL Anxiety → CEA	−0.156**	−0.256**	−0.012 ~ 0.018	−1.523	No Mediation
CI → FL Boredom → CEA	−0.424**	−1.907**	0.009 ~ 0.110	−1.523	*Full Mediation*
L2 Grit → FL Enjoyment → CEA	0.436**	3.366**	0.018 ~ 0.110	1.307	*Full Mediation*
L2 Grit → FL Anxiety → CEA	0.427**	−0.256**	−0.030 ~ 0.018	1.307	No Mediation
L2 Grit → FL Boredom → CEA	0.530**	−1.907**	−0.104 ~ −0.005	1.307	*Full Mediation*

*p < 0.05, **p < 0.01. 95% BootCI indicates the 95% confidence interval calculated from Bootstrap sampling 5,000 times, which is significant if the interval does not include 0.

Regarding the effect of L2 grit on CEA, a similar pattern is observed. The 95% confidence interval for the indirect effect through FL enjoyment does not include zero (0.018 to 0.110). Besides, both path *a* (*L2 grit → FL enjoyment*) and path *b* (*FL enjoyment → CEA*) are significant, while the direct effect (*c’*) is non-significant. This pattern indicates a full mediation effect, supporting the indirect pathway “L2 grit → FL enjoyment → CEA”. Similarly, the indirect effect of L2 grit on CEA via FL boredom is also significant, as the 95% confidence interval does not include zero (−0.104 to −0.005). This result confirms that FL boredom significantly mediates the relationship between L2 grit and CEA, supporting the indirect pathway “L2 grit → FL boredom → CEA”.

### 4.4 Summary

**Figure 2:**
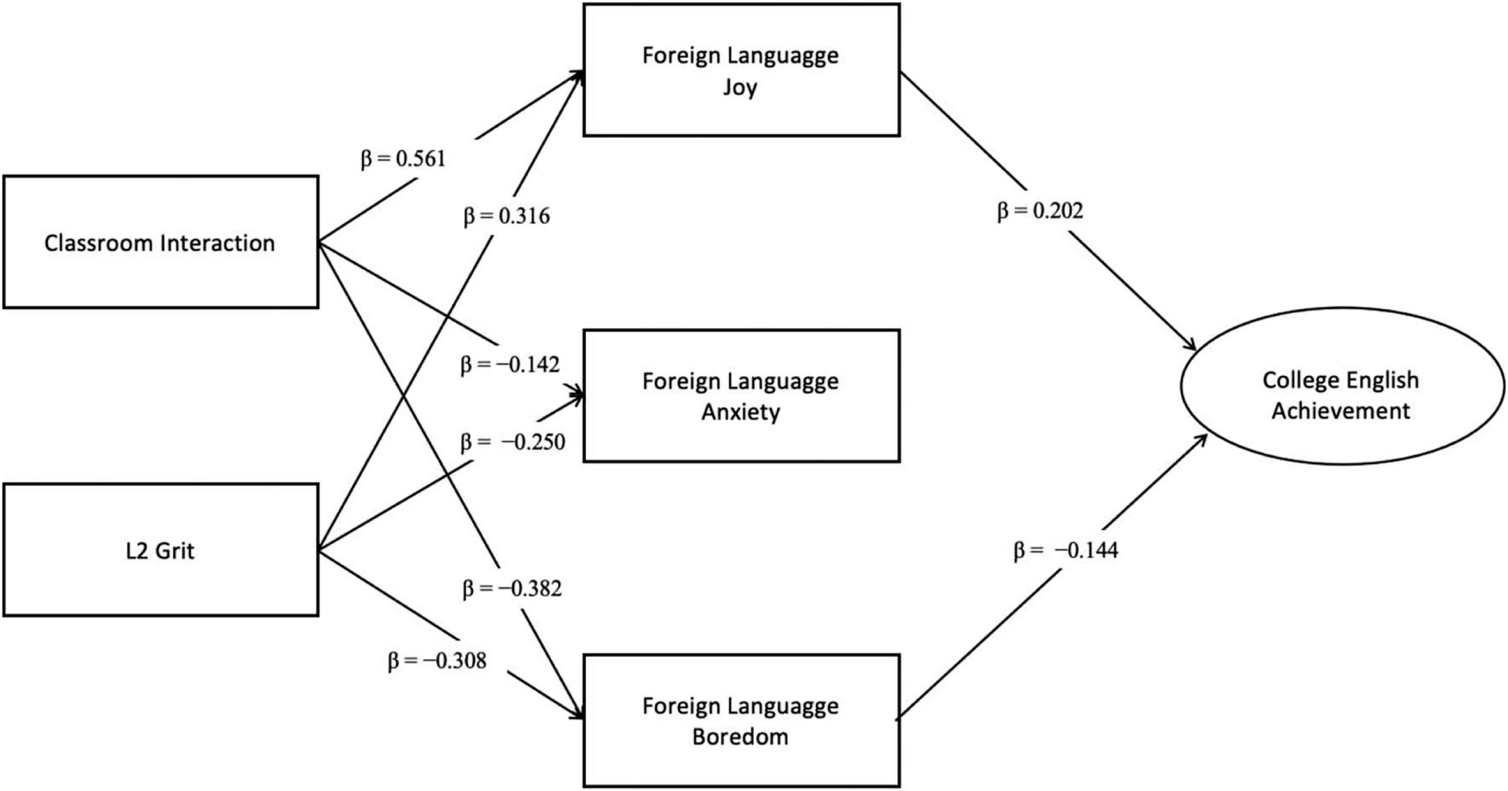
Relationships between CI, L2 grit, FL emotions, and CEA.

According to the data analyzed and presented from 4.1 to 4.3, the results of the empirical model of this study are shown in Figure 2. From the above data, it can be seen that Hypothesis 1 of this study (CI and L2 Grit play a positive predictive role in CEA) has not been verified; however, on the other hand, except for FL anxiety, which does not play a mediating role, FL enjoyment and boredom both play a mediating role in CEA, which makes the Hypothesis 2 of this study to be verified to a certain extent.

## 5 Discussion

This study addresses a critical issue in contemporary college English education. In a system focused mainly on test scores and academic achievement, it examines whether, and through what mechanisms, CI and FL learners’ L2 grit has an influence. The findings have been illustrated in Figure 2. A high-quality interactive classroom and higher levels of L2 grit are associated with greater enjoyment and lower boredom, which in turn are related to better CEA. However, without considering learners’ emotional experiences, these environmental and individual factors do not easily produce measurable achievement gains. All participants were from multiple institutions and cities. Despite this, they were all enrolled in similar College English courses under national curriculum standards. This consistency may have lowered institutional variability in teaching and assessment.

Firstly, contrary to the initial hypotheses, the findings indicate that neither CI nor L2 grit shows a statistically significant direct association with learners’ CEA. This finding is not fully consistent with research suggesting that CI can directly enhance English achievement (e.g., Akpur, 2021; Moskowitz et al., 2022; Lu, 2024), but it does partially support previous studies reporting that the direct effect of L2 grit on language achievement is non-significant (e.g., Khajavy et al., 2021; Wei et al., 2024). It also differs from the negative effect of L2 grit on language achievement reported by Taşpınar and Külekçi (2018). These discrepancies suggest that the associations between CI, L2 grit, and CEA may not be adequately represented by a direct path but may be reflected via other critical psychological variables. Mechanistically, CI appears to be linked with learners’ experiential engagement in the learning process rather than their language outcomes per se (Dai and Wang, 2024). High-quality CI may correspond with learners’ overall perceptions of classroom tasks, teacher-student relationships, and the learning environment. It may also be linked to their emotional experiences and level of engagement (Li T. Y. et al., 2024). However, if such interactions do not internalize into sustained emotional experiences or behavioral engagement, their effect on academic achievement may not be directly observable (Zhang et al., 2025). Similarly, as a relatively stable personality trait, L2 grit may be indirectly associated with academic achievement through learners’ sustained effort, emotional regulation, and behavioral choices within specific learning contexts (Zhang, 2023). Therefore, merely increasing CI or emphasizing the L2 grit alone may be insufficient to directly enhance CEA. Their impact is more likely to manifest indirectly through mediating variables such as emotion and motivation (Wu et al., 2024).

Secondly, this study finds that the effects of CI and L2 grit on CEA are shown through learners’ FL emotions. This points to a dynamic and synergistic relationship among CI, L2 grit, and FL emotions. Specifically, FL enjoyment has a significant positive, full mediating effect between CI and CEA and between L2 grit and CEA. In contrast, FL boredom has a significant negative, full mediating effect. This highlights that external environmental factors are not directly linked to learning outcomes. Instead, these factors relate to learners’ emotional experiences, which are tied to engagement and use of cognitive resources, and ultimately, academic achievement. This result supports the broaden-and-build theory in PP (Fredrickson, 2001; Fredrickson, 2003). A positive CI climate has also been linked to higher levels of positive emotions in learners (Li, 2020). When learners experience enjoyment, they are more likely to see value in tasks and feel in control. This broadens their “thought-action” repertoires and increases participation and persistence. In this study, CI and L2 grit represent the institutional and individual components of the Three Pillars framework. Both are closely connected to FL enjoyment, which also correlates with CEA. This conclusion matches the findings of Dewaele and Dewaele (2017), further supporting the role of positive emotions in connecting environmental factors to learning outcomes. On the other hand, FL boredom is closely tied to perceived task value and control. It is also very sensitive to the quality of CI. High-quality interaction makes tasks more meaningful and increases engagement, reducing boredom. However, once boredom starts, it can lower motivation and attention, negatively impacting academic achievement. In this study, FL boredom consistently shows a significant negative mediating effect, supporting recent research that identifies boredom as an important academic emotion (e.g., Li et al., 2022; Li and Li, 2024). However, one thing needs to be clearly stated: that is, although the present findings support the mediating roles of enjoyment and boredom in linking CI and L2 grit to achievement, alternative causal interpretations should be acknowledged. The proposed direction (emotions to achievement) is theoretically grounded in the broaden-and-build theory (Fredrickson, 2003). Nevertheless, the cross-sectional nature of the data precludes definitive conclusions regarding causal direction. It is equally plausible that academic achievement influences emotional experiences. Students who perform well may experience greater enjoyment and reduced boredom as a result of perceived competence and mastery, whereas lower-performing students may report heightened disengagement or boredom. Such reciprocal relationships are consistent with control-value theory (Pekrun, 2006), which suggests that achievement outcomes and emotional experiences can mutually reinforce each other over time (Pekrun, 2006). Moreover, dynamic systems perspectives in SLA research emphasize that emotions and performance may evolve in a bidirectional and iterative manner rather than following a strictly linear pathway (De Bot et al., 2007). For example, prior achievement may shape self-efficacy and perceived task value, which in turn influence subsequent emotional experiences, creating feedback loops between affect and performance (Kiuru et al., 2020).

Surprisingly, a noteworthy and theoretically informative finding is that FL anxiety does not significantly mediate the relationships between CI or L2 grit and CEA. This result partially diverges from a substantial body of research considering FL anxiety as a core negative emotion in language learning (e.g., Teimouri et al., 2019). Recent studies increasingly suggest that the role of anxiety may be more complex and context-dependent than previously assumed (e.g., Dewaele and Li, 2020; Elahi Shirvan et al., 2021). Meta-analytic evidence indicates that anxiety tends to show a negative overall association with achievement. However, effect sizes are often moderate and highly heterogeneous across educational settings (Teimouri et al., 2019). This implies that contextual moderators may shape its impact. One possible explanation for the non-significant mediating effect observed here is that anxiety levels among non-English-major undergraduates with relatively long learning histories may be comparatively stable and less responsive to variations in classroom climate (Han, 2025). In such cases, anxiety may function more as a background emotional disposition. It becomes less a situationally activated state directly linked to specific classroom dynamics (Han et al., 2022). Moreover, anxiety may influence achievement through alternative pathways not specified in this model. For instance, it may operate through motivational regulation, self-efficacy, beliefs, or learning strategies rather than directly mediating the relationship between contextual or trait variables and performance. Another consideration is that the relationship between anxiety and achievement may not be strictly linear. Some theoretical perspectives suggest that mild or facilitative anxiety can mobilize effort and attention under certain conditions. In contrast, excessive anxiety may impair performance. Such curvilinear or threshold effects, however, were not tested in this study. Similarly, potential effects, such as anxiety×L2 grit or anxiety×CI, were beyond the scope of this analysis. Therefore, the absence of anxiety should not be interpreted as evidence that anxiety is irrelevant to language learning. Rather, it suggests that, within the hierarchical mechanism specified here, anxiety may not function as the primary proximal conduit linking CI and L2 grit to academic achievement. Future research adopting longitudinal, multilevel, or nonlinear modeling approaches may help clarify the conditions under which anxiety exerts direct, indirect, or interactive influences on learning outcomes.

Overall, this study identifies the facilitating role of positive FL emotions and the inhibitory role of negative emotions in relation to academic achievement, consistent with prior research (e.g., Dewaele and Dewaele, 2017). By integrating CI, L2 grit, FL emotions, and CEA within a single analytical framework, this study elucidates, from the perspective of PP, the synergistic “from environment to personality and to emotion” mechanism and underscores the critical role of emotional experience in linking instructional context and learning outcomes. The results suggest that academic achievement may be enhanced when a positive CI climate and learner traits are effectively linked through emotional experiences.

## 6 Implications

The pedagogical implications of this study should be interpreted in light of its core empirical finding: CI and L2 grit did not exert direct effects on CEA but operated indirectly through learners’ emotional experiences, particularly enjoyment and boredom. This pattern suggests that instructional interventions targeting emotional processes may be more effective than those focusing solely on behavioral participation or individual effort.

First, the full mediating role of FL enjoyment highlights a key point. Enhancing the quality of CI fosters positive emotional experiences. Therefore, teachers should not treat interaction as an end in itself. Instead, they should use it to cultivate enjoyment through supportive feedback, meaningful communicative tasks, and a psychologically safe classroom climate. Simply increasing the frequency of interaction, without attention to emotional quality, may have a limited impact on achievement.

Second, the significant negative mediating role of boredom underscores a clear need. Reducing emotional disengagement is important in exam-oriented college English classrooms. Boredom was a key emotional pathway linking both CI and L2 grit to achievement. Instruction should prioritize task variety, optimal challenge, and perceived task value. From this perspective, reducing boredom may represent not only an affective concern but also a potential avenue for improving academic outcomes.

Third, the absence of direct effects of CI and L2 grit on achievement is notable. Encouraging perseverance or participation alone may not be enough to enhance learning outcomes. Teachers should focus on how students emotionally experience sustained effort and classroom demands. Instructional designs that help students see effort as meaningful and emotionally rewarding are more likely to translate persistence into achievement gains.

## 7 Conclusion

This study recruited second-year non-English-major undergraduates in China as participants and employed path analysis and mediation analysis to examine the predictive effects of CI and L2 grit on CEA, as well as the mediating role of FL emotions, including FL enjoyment, anxiety, and boredom. The results indicated that neither CI nor L2 grit had a direct effect on CEA. However, both exerted indirect effects through FL emotions: FL enjoyment served as a positive mediator between CI, L2 grit, and CEA, whereas FL boredom functioned as a negative mediator in these relationships.

Admittedly, this study has several limitations. First, the cross-sectional design precluded conclusions about causal or developmental relationships among variables. Second, as stated in 3.2, participants were recruited through convenience sampling from three universities within two cities, which concerns the sampling structure. Although participants were recruited using a standardized curriculum and textbook, institutional variation was not explicitly modeled. In addition, some relevant learner background variables (e.g., prior English proficiency, majors or family background) were not collected, which may introduce unobserved heterogeneity in the data. Third, the use of a composite achievement score may introduce potential criterion contamination, as CI and emotional variables could directly influence process-based components such as participation and attendance. This may partially inflate associations between predictors and achievement. Future research should consider using more independent outcome measures, such as standardized test scores or separate analyses of summative and formative assessment components, to further strengthen causal interpretation. Lastly, this study only explored FL enjoyment, anxiety and boredom, and had not yet been designed for other emotions, and subsequent research is worthwhile to go deeper.

## Data Availability

The raw data supporting the conclusions of this article will be made available by the authors, without undue reservation.
